# Gender trends in authorships and publication impact in Academic Radiology—a 10-year perspective

**DOI:** 10.1007/s00330-021-07928-4

**Published:** 2021-05-19

**Authors:** Isabel Molwitz, Jin Yamamura, Ann-Kathrin Ozga, Ilka Wedekind, Thai-An Nguyen, Liesa Wolf, Minobu Kamo, Jing Zhao, Elif Can, Sarah Keller

**Affiliations:** 1 University Medicine Hamburg-Eppendorf (UKE), Martinistraße 52, 20246 Hamburg, Germany; 2 Charité Universitaetsmedizin Berlin corporate member of Freie Universität Berlin, Humboldt-Universität zu Berlin, and Berlin Institute of Health, Charitéplatz 1, 10117 Berlin, Germany; 3 St. Luke’s International Hospital, 9-1 Akashi-cho, Chuo-ku, Tokyo, 104-8560 Japan

**Keywords:** Authorship, Female, Journal impact factor, Bibliometrics, Publishing/statistics

## Abstract

**Objectives:**

To analyze the development of publication numbers of female authors in high-, medium-, and low-impact radiological journals.

**Methods:**

In this bibliometric analysis, gender of the first (FA) and senior author (SA) was assigned to all original research articles and reviews, published in 10 high-, medium-, and low-impact radiological journals in 2007/8 and 2017/18. The adjusted event rate (AER) and adjusted odds ratio (AOR) were calculated using mixed logistic and multinomial logistic regression models to assess and compare female publications according to impact factor, journal, author position, and combination.

**Results:**

The proportion of female FA and female SA in *N* = 6979 (2007/2008) and *N* = 7383 (2017/2018) articles increased to 29.1% and 16.1% in 2017/2018, respectively. While most female authorships were continuously observed in medium-impact journals, the strongest increase occurred for both female FA (AOR 2.0; *p* < .0001) and SA (AOR 2.1; *p* < .0001) in low-impact journals. Female SA published significantly more often in a low- (AOR 1.5) or medium- (AOR 1.8) than in a high-ranking journal. Among the high-ranking journals, female FA published most frequently in *European Radiology* (32.4%; 95% CI [29.3–35.8]; *p* < .0001), female SA in *Investigative Radiology* (15.9%; 95% CI [13.7–18.4]; *p* < .0001). Male same-sex authorships decreased (AOR 0.9), but remained at least twice as common as all-female or mixed authorships.

**Conclusion:**

The increase in female authorship is reflected in all impact areas. Female FA and SA increased most in low-ranking journals but are most common in medium-ranking journals. Female SA remain rare, especially in high impact journals.

**Key Points:**

*• Compared to the proportion of female radiologists worldwide, female senior authors are underrepresented in all impact areas, in particular in high-impact journals.*

*• Among the included high-ranking radiological journals, female first authors and senior authors were strongest represented in European Radiology and Investigative Radiology, while across all impact areas they mostly published in medium-ranking journals.*

*• Female author combinations were more frequent in low- and medium- than in high-ranking journals, whereas male author combinations remained more common than female senior author collaborations in all impact areas.*

## Introduction

Nowadays, female students make up for more than half of all medical students in European countries, as the UK, Sweden, or Germany [1], and in the USA [2]. The rate of female doctors in these countries ranges well within the gender balanced zone of 40–60% [1]. Concerning radiologists, 33.5% worldwide are female; in the age cohort of those under 35 years, the proportion of female radiologists ranges up to 48.5% [3].

However, although women are even more likely than men to begin academic careers in medicine after training, they lag behind their male counterparts in obtaining levels of rank such as faculty senior positions [4–6]. A major factor influencing an academic advancement is the h-index, which is dependent on scholarly productivity based on peer-reviewed scientific publications and the number of citations. Fewer high-ranked publications might lead to less citations and thus poorer chances of promotion to senior positions. Systematic analyses of female authorships were successful in revealing the unequal distribution of leading author positions in radiology, while at the same time demonstrating a positive trend in development [7, 8].

The latest analyses however ended in 2016 and focused on high-ranking radiological journals such as *European Radiology* (Eur Radiol), *Radiology*, and the *American Journal of Radiology* (AJR) [7–10]. The aim of this study is to address this gap and to generate a comparison of the publication numbers of female authors from the last 10 years, between 2007/2008 and 2017/2018, in ten representative radiological journals of varying impact factors. Furthermore, the frequency of same-sex and mixed-sex first and senior author co-operations was examined to generate insights into gender-specific collaboration of junior and senior faculty members.

## Materials and methods

**Fig. 1:**
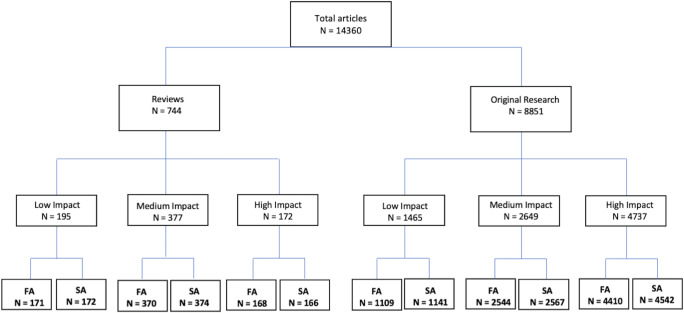
Flowchart of all included publications for 2007/2008 and 2017/2018 independent of gender. All included publications were classified as original research articles, reviews, and others (e.g., letter to the editor). The number of the first and senior authors (male and female) which were successfully assigned to one gender is given. Abbreviations: FA first author, SA senior author

An institutional review board exemption was obtained for this retrospective study. Ten radiological journals of high, medium, and low impact according to the Clarivate analytics web of science’s journal citation reports for “Radiology, Nuclear Medicine, and Medical Imaging” were chosen. Care was taken to include only representative, general radiological journals and no subspeciality journals, e.g., for cardiovascular imaging, and to include journals from the European, American, and Asian continent to take potentially varying developments between journals from different regions into account. Also, as far as feasible journals were only included, if their impact factor did not change between the defined low impact area (impact factor range: 1.9 to 1.5), medium impact area (range: 3.2 to 2.1), or high impact area (range: 7.6 to 3.9) over the course of the study period (2007/2008 to 2017/2018). We finally included the following journals in our analysis: high impact journals—*Radiology*, *Investigative Radiology* (Invest Radiol), Eur Radiol, and *Magnetic Resonance in Medicine* (MRM); medium impact journals—AJR, *European Journal of Radiology* (EJR), and *Clinical Radiology* (Clin Radiol); lower impact journals—*Fortschritte auf dem Gebiet der Röntgenstrahlen und bildgebenden Verfahren* (Rofo), *Acta radiologica* (Acta Radiol), and *Japanese Journal of Radiology* (Jpn J Radiol). Of these, only MRM changed from medium to high impact between 2007 and 2008 and Clin Radiol from low to medium impact from 2007/2008 to 2017/2018. Both were assigned to their impact area based on the impact factor in 2017/2018. All publications of 2007, 2008, 2017, and 2018 were listed and classified as original research articles (ORA), reviews, and others (e.g., editorials, pictorial essays, letters to the editor). Only ORAs and reviews were included into further analysis, as authorships of letters to the editor, comments, etc. were considered to be less relevant for a scientific career. The full names of the first authors (FA) and senior authors (SA) were obtained from all included ORAs and reviews. All first names were manually assigned to their supposed gender by the German authors IW, LW, and TN. Asian names were re-evaluated by native speakers (Japan, Korean, Vietnamese, Chinese). If available, ORCID IDs were used to verify the identification. In the case of gender-ambiguous first names (about 40%), several steps to ascertain high assignment were taken. First, further information concerning the author was searched by common and scientific search engines as “google” and “research gate.” If only author initials were provided, other publications of the same research group as well as pictures, gender-specific pronouns, and CVs on the webpages of the authors’ institutes were analyzed. Lastly, if the sex was still uncertain, it was classified as unknown. An overview of the number of all included publications, of the number of article types, and of female FA and SA depending on article type and impact factor is given in the flowchart (Fig. 1).

To assess an under- or overrepresentation of female authors in the included international journals, female author proportions were compared to the number of female radiologists, as given by a study of Cater et al [3] from 2018 who made inquiries about the members of radiological societies from North, Central, and South America, Europe, Asia, Africa, and Oceania. Mean rate of female radiologists worldwide was thus given with 33.5%, with a range of 27.2% (in the USA) to 85% (in Thailand) and proportions that ranged between 28.8 and 68.9% for European radiological societies [3]. As the first authors are more likely to be junior faculty members, female FA were additionally put in relation to the mean number of radiologists of 35 years or younger (48.5%) [3].

### Statistical analysis

Logistic mixed regression models were used to analyze any difference in the percentages of female and male authors. The outcome variable was indicating whether the FA or SA of the publication was female. FA variable and SA variable were the same for ORAs and reviews. If not indicated, otherwise all results apply thus for authors of ORAs and reviews taken together. Since for each publication two observations were given (i.e., one information for the first author and one for the senior author), a random effect was included in the model indicating the publication identification. As fixed effects, year, journal, impact factor, and the variable indicating whether the observations belonged to the first or senior author were considered in the model as well as interactions between the fixed effects variables. Within this model, an observation was defined missing if the gender for the first and senior authorship was unknown. A publication could be included in the analysis if at least for one authorship (either first or senior, or both) gender was given. A multinomial logistic regression model was used to evaluate any differences between percentages of the four possible author combinations (i.e., male-both, female-both, male FA-female SA, female FA-male SA). Thus, the categorical outcome variable consisted of these four groups. The year and journal as well as the corresponding interaction term were included as independent variables in the model. Observations from 2007 and 2008 and 2017 and 2018 were considered as two time points in the analysis: 2007/2008 and 2017/2018. In the multinomial logistic regression model, missing observations were defined as unknown sex of either the FA or SA (or both). Adjusted event rates (AER) and adjusted odds ratio (AOR) along with 95% confidence intervals are reported throughout the manuscript, i.e., odds ratios of a comparison are adjusted for all other independent variables in a model (model-based estimates). The odds ratio was used to describe differences between time points or between the publication shares of two groups, e.g., female first versus male first authors. All analyses and thus *p*-values are considered descriptive due to the explorative study design (i.e., no adjustment for multiple testing was conducted). Statistic software R (Version 3.5.1) was used. Statistical analyzes were conducted by AO.

## Results

### Overview

A total of 6978 publications were extracted for 2007/2008 and 7382 for 2017/2018. The gender of FA was successfully determined for 4163 publications (59.7%) in 2007/2008 and 5650 (76.5%) in 2017/2018. The gender of SA was determined in 4181 publications (59.9%) in 2007/2008 and 5756 (78.0%) in 2017/2018. In total, 218 (30.7%) female FA and 159 (22.3%) female SA were found for review articles, 2379 (29.5%) and 1395 (16.9%) were female FA and SA, respectively, in ORA. Publication shares of female authors were thus lowest for SA in original research papers.

### Development of publication shares between 2007/2008 and 2017/2018

**Fig. 2.**
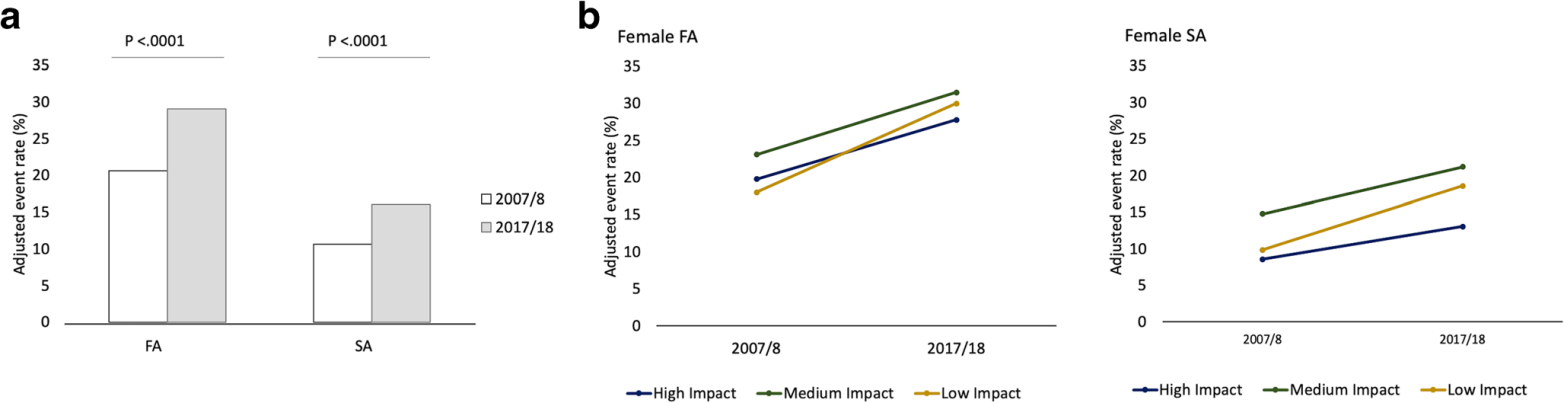
Development of publication numbers of female FA and SA between 2007/2008 and 2017/2018. **a** Increase in total publication shares of female FA (20.6 to 29.1%; *p* < .0001) and SA (10.7 to 16.1%; *p* < .0001). **b** Increase in the publication shares stratified by impact factor over a 10-year interval. Low impact journals showed the steepest increase in publication figures for female FA and SA over the course of 10 years (adjusted odds ratio 2.0; *p* < .0001). The proportion of female FA and SA was consistently highest in medium impact journals (FA: 23.1 to 31.5%; SA: 14.8 to 21.2%). Abbreviations: FA first author, SA senior author

The proportion of leading female authors (FA and SA together) rose from 1575 (16.1%) in 2007/2008 to 2938 (23.0%) in 2017/2018 (AOR 1.6 [95% CI 1.4–1.7]; *p* < .0001). Of these, the publication numbers of female FA and SA increased from 1006 (20.6%) to 1813 (29.1%) and from 569 (10.7%) to 1125 (16.1%), respectively (Fig. 2a). Both female FA and SA proportions were thus smaller than the rate of female radiologists worldwide (33.5%) as provided by Cater et al [3]. Female SA shares were also lower than the smallest described national female radiologist rate (27.2% in the USA) [3].

### Female authorships stratified by impact factors

Stratified by impact factors, the proportion of female authors (FA and SA together) increased significantly in all impact areas (*p* < .0001), with low impact journals showing the greatest increase (14.6 to 24.8%), followed by medium (19.5 to 26.8%) and high impact journals (14.6 to 20.8%). The increase was even higher in low impact journals broken down to female FA (AOR 2.0; [95% CI 1.5–2.6]; *p* < .0001) and SA (AOR 2.1 [95% CI 1.5–2.9]; *p* < .0001) (Fig. 2b). Nevertheless, the share of all-female leading authorships (FA and SA together) was consistently highest in medium impact journals (2007/2008: 19.5% [95% CI 17.9–21.3]; 2017/2018: 26.8% [95% CI 25.0–28.7]) (Fig. 2b; for absolute numbers, please see Table 1). While female SA were clearly more common in the medium impact area (AOR medium vs. high impact 1.8 [95% CI 1.5–2.1]; *p* < .0001) or low impact area (AOR low vs. high 1.5 [95% CI 1.2–1.9]; *p* < .0001) than in high impact journals, the frequency of female FA between the impact areas differed less (AOR between 0.93 and 1.18) (Table 2).

**Table 1:** Change of all-female authorships from 2007/2008 to 2017/2018 and separately for journals of high, medium, and low impact factor

	2007/2008			2017/2018				
	Total observations	Female observations	AER [95% CI]	Total observations	Female observations	AER [95% CI]	AOR [95% CI]	Descriptive *p*-value
Overall	8344	1575	16.1 [15.1–17.2]	11406	2938	22.99 [22.0–24.1]	1.6 [1.4–1.7]	< .0001
High IF	4608	791	14.6 [13.4–15.8]	6380	1499	20.8 [19.5–22.0]	1.5 [1.4–1.7]	< .0001
Medium IF	2796	622	19.5 [17.9–21.3]	3145	923	26.8 [25.0–28.7]	1.5 [1.3–1.7]	< .0001
Low IF	940	162	14.6 [12.4–17.2]	1881	516	24.8 [22.6–27.2]	1.9 [1.5–2.4]	< .0001

Logistic mixed model. Abbreviations: AER adjusted event rate, AOR adjusted odds ratio, IF impact factor, CI confidence interval

**Table 2:** Proportions of female authors separated by impact range and year

	High IF			Medium IF			Low IF			Adjusted odds ratio (AOR [95% CI])					
	Total observ.	Fem. observ.	AER [95% CI]	Total observ.	Fem. observ.	AER [95% CI]	Total observ.	Fem. observ.	AER [95% CI]	Medium–high AOR [95% CI]	Des. *p*-value	Low–high AOR [95% CI]	*p*-value	Low–medium AOR [95% CI]	Des. *p*-value
Overall	10988	2290	18.0 [17.1–19.0]	5941	1545	23.2 [21.9–24.5]	2821	678	21.2 [19.4–23.0]	1.4 [1.2–1.5]	< .0001	1.2 [1.1–1.4]	.0013	0.9 [0.8–1.0]	.142
2007/2008	4608	791	14.6 [13.4–15.8]	2796	622	19.5 [17.9–21.3]	940	162	14.6 [12.4–17.2]	1.4 [1.2–1.7]	< .0001	1.0 [0.7–1.4]	1.00	0.7 [0.5–1.0]	.0199
2017/2018	6380	1499	20.8 [19.5–22.0]	3145	923	26.8 [25.0–28.7]	1881	516	24.8 [22.6–27.2]	2.1 [1.6–2.9]	< .0001	1.3 [1.0–1.5]	.0106	0.9 [0.7–1.1]	.7453
2007–2018															
Female FA	5462	1510	24.2 [22.8–25.6]	2957	902	27.3 [25.4–29.2]	1394	407	25.8 [23.3–28.5]	1.2 [1.0–1.4]	.0603	1.1 [0.9–1.4]	.8655	0.9 [0.7–1.2]	.9460
Female SA	5526	780	11.0 [10.1–12.0]	2984	643	18.0 [16.4–19.6]	1427	271	15.5 [13.6–17.7]	1.8 [1.5–2.1]	< .0001	1.5 [1.2–1.9]	< .0001	0.8 [0.7–1.1]	.3865

Logistic mixed model including adjusted odds ratio (AOR) and adjusted event rate (AER) with 95% confidence interval (CI). Abbreviations: IF impact factor, Total observ. total observations, Fem. observ. female observations, Des. *p*-value descriptive *p*-value, FA first author, SA senior author

### Female authorships stratified by the journal

**Fig. 3:**
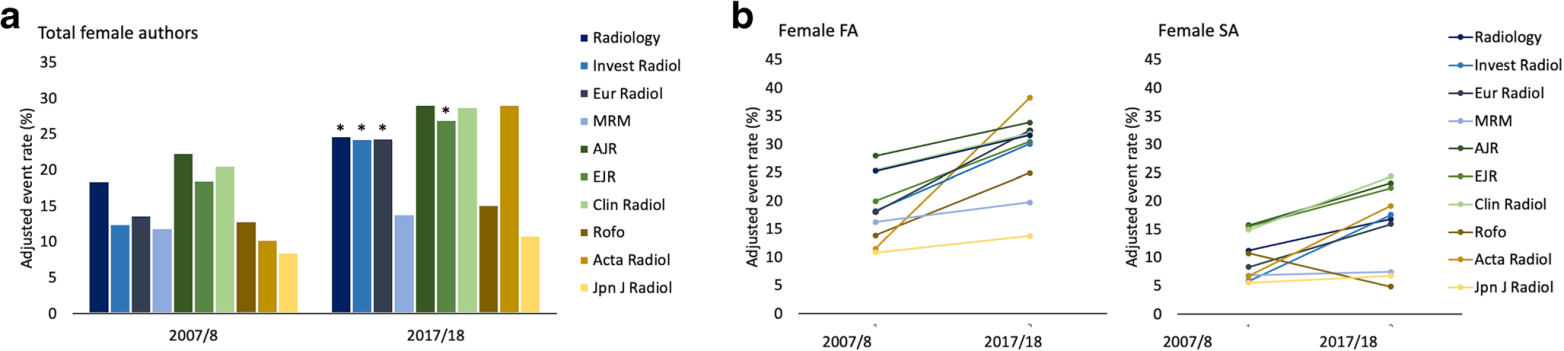
Development of female authorship between 2007/2008 and 2017/2018, separated by journal. **a** Over the course of 10 years, female authorships increased significantly in *Radiology* (adjusted odds ratio (AOR) 1.5 [95% CI 1.2–1.7]; *p* < .0001), Invest Radiol (AOR 2.3 [95% CI 1.5–3.3]; *p* < .0001), Eur Radiol (AOR 2.0 [95% CI 1.7–2.5]; *p* < .0001), and EJR (AOR 1.6 [95% CI 1.2–1.7]; *p* < .0001). **b** Changes in author shares between 2007/2008 and 2017/2018 grouped by FA and SA and sub-grouped by journal. Eur Radiol was the only journal to show an increase in both female FA (18.1 to 32.4%, *p* < .0001) and SA (8.3 to 15.9%; *p* < .0001) after starting in 2007/2008 with comparably low shares. Abbreviations: FA first author, SA senior author

In the 10-year comparison, the publication shares of female lead authors (FA and SA together) increased significantly in Invest Radiol (12.5 to 24.3%, AOR 2.3 [95% CI 1.5–3.3]), Eur Radiol (13.7 to 24.4%, AOR 2.0 [95% CI 1.7–2.5]), EJR (18.6 to 27.0%, AOR 1.6 [95% CI 1.2–1.7]), and *Radiology* (8.5 to 24.7%, AOR 1.5 [95% CI 1.2–1.7]) (Fig. 3a).

With respect to female FA, the increase was significant only for Eur Radiol (18.1 to 32.4%, AOR 2.2 [95% CI 1.7–2.8]; *p* < .0001). The highest publication shares of female FA in 2017/2018 were found for Acta Radiol (38.2%) followed by AJR (33.9%), and Eur Radiol (32.4%). Eur Radiol also demonstrated the greatest proportion of female FA (32.4%) within the high impact area in 2017/2018 (Fig. 3b, Table 3). The lowest proportion of female FA in all journals from the high or medium impact area and also the lowest increase over 10 years (16.2 to 19.7%, AOR 1.3 [95% CI 1.0–1.7]; *p* = .09) was documented for MRM. Across all impact areas, Jpn J Radiol showed the lowest proportion of female FA associated with one of the lowest increases of female FA in 10 years (10.8 to 13.8%, AOR 1.3 [95% CI 0.7–2.4]; *p* = .38) (Fig. 3b, Table 3).

**Table 3:** Change of all-female authorships from 2007/2008 to 2017/2018 separated by journal and author position

Journal title	2007/2008			2017/2018			2007/2008 vs. 2017/2018	
	Total observations	Female observations	AER [95% CI]	Total observations	Female observations	AER [95% CI]	AOR [95% CI]	Descriptive *p*-value
First author								
*Radiology*	844	239	25.3 [22.1–28.7]	947	323	31.6 [28.3–35.1]	1.4 [1.1–1.7]	.0076
Invest Radiol	219	47	18.2 [13.4–24.2]	184	60	30.1 [23.2–38.0]	1.9 [1.2–3.2]	.0105
Eur Radiol	615	131	18.1 [15.1–21.5]	1035	360	32.4 [29.3–35.8]	2.2 [1.7–2.8]	< .0001
MRM	617	120	16.2 [13.4–19.5]	1001	230	19.7 [17.2–22.6]	1.3 [1.0–1.7]	.0900
AJR	736	226	27.9 [24.4–31.7]	757	274	33.9 [30.1–37.9]	1.3 [1.0–1.7]	.0268
EJR	486	112	20.0 [16.4–24.1]	690	229	30.5 [26.7–34.5]	1.8 [1.3–2.4]	.0002
Clin Radiol	248	71	25.4 [19.9–31.8]	381	131	31.9 [26.8–37.4]	1.4 [0.9–2.0]	.1170
ROFO	177	30	13.9 [9.5–19.9]	111	31	24.9 [17.1–34.7]	2.1 [1.1–3.9]	.0270
Acta Radiol	28	4	11.5 [4.0–29.0]	360	144	38.2 [32.7–44.2]	4.8 [1.5–15.5]	.0091
Jpn J Radiol	193	26	10.8 [7.2–16.0]	184	31	13.8 [9.4–19.6]	1.3 [0.7–2.4]	.3815
Senior author								
*Radiology*	847	116	11.2 [9.2–13.6]	903	182	16.8 [14.4–19.6]	1.6 [1.2–2.1]	.0009
Invest Radiol	222	17	5.9 [3.6–9.50]	188	39	17.6 [12.6–24.0]	3.4 [1.8–6.6]	.0002
Eur Radiol	617	66	8.3 [6.4–10.7]	1087	206	15.9 [13.7–18.4]	2.1 [1.5–2.9]	< .0001
MRM	627	55	6.8 [5.2–9.0]	1035	99	7.4 [6.0–9.2]	1.1 [0.8–1.6]	.6046
AJR	742	140	15.7 [13.2–18.7]	767	202	23.2 [20.1–26.7]	1.6 [1.2–2.1]	.0005
EJR	485	91	15.5 [12.4–19.1]	709	180	22.3 [19.1–25.9]	1.6 [1.1–2.2]	.0052
Clin Radiol	254	45	14.9 [10.9–20.0]	411	113	24.4 [20.1–29.2]	1.8 [1.2–2.8]	.0052
ROFO	170	23	10.8 [7.0–16.3]	111	7	4.8 [2.2–10.1]	0.4 [0.2–1.1]	.0671
Acta Radiol	24	2	6.7 [1.5–25.0]	362	81	19.2 [15.2–23.9]	3.3 [0.7–15.7]	.1331
Jpn J Radiol	193	14	5.5 [3.2–9.4]	183	16	6.8 [4.0–11.1]	1.2 [0.6–2.7]	.5913

Multinominal logistic regression model. Abbreviations: AER adjusted event rate, AOR adjusted odds ratio, CI confidence interval

The highest publication shares of SA across all impact areas in 2017/2018 were found in medium-ranked journals, with the highest proportions in Clin Radiol (24.4%) followed by AJR (23.2%) and EJR (22.3%) (Fig. 3b). As with female FA, the increase of female SA in all included journals was only significant for Eur Radiol (8.3 to 15.9%, AOR 2.1 [95% CI 1.5–2.9]; *p* < .0001). Within the high impact area in 2017/2018, publication shares of female SA were comparable (*Radiology* 16.8%, Invest Radiol 17.6%, Eur Radiol 15.9%) with the exception of MRM (7.4%) (Table 3). As for female FA, amongst the high- and medium-ranked journals, MRM also showed the smallest increase in female SA (6.8 to 7.4%, AOR 1.1 [95% CI 0.8–1.6]; *p* = .6046). Across all impact areas, the increase of female SA in MRM was second last to Rofo (10.8 to 4.8%, AOR 0.4 [95% CI 0.2–1.1]; *p* = .0671), for which lowest publication shares of female SA in 2017/2018 were found (4.8%) (Fig. 3b).

### Publication shares of mixed- and same-sex authorships

**Fig. 4:**
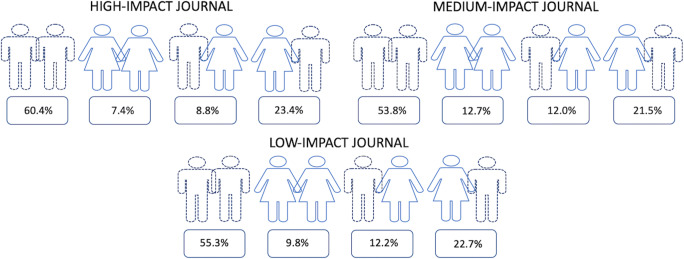
Publication co-operations between female and male leading authors in 2017/2018. The highest publication shares (adjusted event rate %) were found for same-sex male first authors and male senior authors. The lowest publication shares were found for same-sex female authors

In 2017/2018 in each, the high, medium, and low impact areas, same-sex male authorships (60.4%, 53.8%, 55.3%) were at least four times more common than same-sex female authorships (7.4%, 12.7%, 9.8%) or mixed-sex female SA authorships (8.8%, 12.0%, 12.2%). Same-sex male authorships also remained twice as common as mixed-sex male SA authorships (23.4%, 21.5%, 22.7%) and the latter were still the double of same-sex female authorships. In 2017/2018, with the exception of the medium impact area, mixed-sex female SA authorships (8.8%, 12.0%, 12.2%) were found slightly more frequently than female same-sex authorships (Fig. 4). For absolute values, please see Table 4.

**Table 4:** Publication shares of mixed- or same-sex authors cooperations according to journal in 2017/2018

2017/2018	Observations and percentages									Probabilities between author combinations (AOR [95% CI])							
	Total observ.	♂♂		♀♀		♀♂		♂♀		♂♂ vs. ♀♀	Des. *p*-value	♀♂ vs. ♀♀	Des. *p*-value	♀♂ vs. ♂♀	Des. *p*-value	♂♂ vs. ♀♂	Des. *p*-value
		Group observ.	AER [95% CI]	Group observ.	AER [95% CI]	Group observ.	AER [95% CI]	Group observ.	AER [95% CI]								
*Radiology*	858	479	55.8 [52.4–59.2]	102	11.9 [9.7–14.1]	202	23.5 [20.7–26.4]	75	8.7 [6.8–10.7]	1.5 [1.5–1.7]	< .0001	1.1 [1.1–1.2]	< .0001	1.2 [1.1–1.2]	< .0001	1.4 [1.3–1.5]	< .0001
Invest Radiol	184	101	54.9 [47.6–62.2]	14	7.6 [3.7–11.5]	46	25.0 [18.6–31.4]	23	12.5 [7.6–17.4]	1.6 [1.4–1.8]	< .0001	1.2 [1.1–1.3]	.0003	1.1 [1.0–1.3]	.0314	1.4 [1.1–1.6]	< .0001
Eur Radiol	1008	566	56.2 [53.0–59.3]	85	8.4 [6.7–10.2]	258	25.6 [22.9–28.3]	99	9.8 [8.0–11.7]	1.6 [1.5–1.7]	< .0001	1.2 [1.1–1.2]	< .0001	1.2 [1.1–1.2]	< .0001	1.4 [1.3–1.5]	< .0001
MRM	991	692	69.8 [66.9–72.7]	25	2.5 [1.5–3.5]	205	20.7 [18.1–23.3]	69	7.0 [5.4–8.6]	2.0 [1.9–2.1]	< .0001	1.2 [1.2–1.2]	< .0001	1.2 [1.1–1.2]	< .0001	1.6 [1.5–1.8]	< .0001
AJR	752	387	51.5 [47.8, 55.1]	107	14.2. [11.7–16.8]	165	21.9 [18.9, 25.0]	93	12.4 [10.0,14.8]	1.5 [1.4, 1.6]	< .0001	1.1 [1.0, 1.1]	.0042	1.1 [1.0, 1.2]	.0002	1.3 [1.2, 1.5]	< .0001
EJR	682	371	54.4 [50.6–58.2]	88	12.9 [10.3–15.5]	137	20.1 [17.0–23.2]	86	12.6 [10.1–15.2]	1.5 (1.4–1.6]	< .0001	1.1 [1.0–1.1]	.0088	1.08 [1.0–1.1]	.0056	1.4 [1.3–1.5]	< .0001
Clin Radiol	350	176	50.3 [44.9–55.6]	43	12.3 [8.8–15.8]	74	21.1 [16.8–25.5]	57	16.3 [12.3–20.2]	1.5 [1.3–1.6]	< .0001	1.1 [1.0–1.2]	.0261	1.1 [1.0–1.1]	.4499	1.3 [1.2–1.5]	< .0001
ROFO	111	74	66.7 [57.7–75.6]	1	0.9 [NA–2.7]	30	27.0 [18.6–35.5]	6	5.4 [1.1–9.7]	1.9 (1.7–2.2]	< .0001	1.3 [1.2–1.5]	< .0001	1.2 [1.1–1.4]	.0003	1.5 [1.2–1.9]	< .0001
Acta Radiol	346	169	48.8 [43.5–54.2]	39	11.3 [7.9–14.7]	99	28.6 [23.8–33.5]	39	11.3 [7.9–14.7]	1.5 [1.3–1.6]	< .0001	1.2 [1.1–1.3]	< .0001	1.2 [1.1–1.3]	< .0001	1.2 [1.1–1.4]	.0003
Jpn J Radiol	180	139	77.2 [71–83.5]	4	2.2 [0–4.4]	26	14.4 [9.2–19.7]	11	6.1 [2.5–9.7]	2.1 [1.9–2.3]	< .0001	1.1 [1.1–1.2]	.0005	1.1 [1.0–1.2]	.0687	1.9 [1.6–2.2]	< .0001

Multinominal logistic regression model. Abbreviations: AER adjusted event rate, AOR adjusted odds ratio, CI confidence interval, ♂♂ female same-sex authorships, ♀♀ male same-sex authorships, ♀♂ female first author with male last author, ♂♀ male first author with female last author

With regard to the development over time, the shares of same-sex male combinations taken together for all impact areas decreased slightly between 2007/2008 and 2017/2018 (AOR 0.91 [95% CI 0.9–0.9]; *p* < .0001) and the shares of female same-sex combinations increased slightly (AOR 1.04 [95% CI 1.0–1.1]; *p* = .0002). In particular, the female same-sex frequency increased most in the medium impact area (7.4 to 12.7%, AOR 1.1 [95% CI 1.0–1.1]; *p* < .0001), while for male same-sex combinations, it decreased most for the low impact area (69.2 to 55.3%, AOR 0.9 [95% CI 0.8–0.9]; *p* < .0001). The estimated percentages of both mixed authorship combinations developed similarly in all impact areas and changed between 2 and 5% at maximum. Out of all journals, Jpn J Radiol showed the highest proportions of same-sex male combinations in 2017/2018 (77.2% [95% CI 71.0–83.5]) (Table 4). Regarding high- and medium impact journals, AJR showed the highest percentage (14.2% [95% CI [11.7–16.8]), and MRM (2.5% [95% CI 1.5–3.5]) the lowest percentage of female same-sex authorships in 2017/2018.

## Discussion

This is the first study which investigated and compared female author shares between radiological journals of low, medium, and high impact factor. The main findings of this study were that (a) apart from a positive trend in the publication shares of female authors in all impact areas between 2007/2008 and 2017/2018—which however was significant only in Eur Radiol, EJR, Invest Radiol, and *Radiology*—(b) women were differently represented depending on the impact areas. Low-ranked journals showed the highest increase of female authors, while female author proportions were consistently highest in medium impact journals. Especially, female SA remained underrepresented in all impact areas, notably in high-ranked journals. (c) Same-sex male authorships in 2017/2018 were still four times more common than same-sex female or mixed-sex female SA authorships and twice as common as mixed-sex male SA authorships.

The proportion of female FA (29.1%) and SA (16.1%) taken together for all impact areas in 2017/2018 as ascribed in this study is lower than in previous studies. For example, in an analysis from 2011 to 2015 of the medium- to high-ranked journals *Radiology*, *Academic Radiology*, and AJR, Campbell et al [7] attributed 30% of FA and 25% of SA to women. A study of the four medium- to high-ranking journals *Radiology*, EJR, *Journal of Computer Assisted Tomography*, and AJR described a share of 31.5% FA and 22.1% SA in 2014 [10]. For *European Radiology*, 35.0% FA and 18.0% SA were described for 2016 [9]. These differences may be due to the fact that our study examined a higher range of representative journals and thus a higher number of articles from all impact areas. In addition, our analysis with data from the years 2017/2018 is more up to date compared to previous studies that examined publication dates until 2016 [9].

To judge about female representation based on the number of described female authorships, the number of female radiologists as provided in the literature was considered. The rate of 17% female SA demonstrated an apparent underrepresentation compared to even the country with the lowest female radiologist rates (USA 2018: 27.2%) [3]. Female FA would be underrepresented compared to the mean rate of female radiologists worldwide (33.5%) [3]. If comparing female FA to the mean rate of female radiologists aged 35 years or younger (48.5%), as FA are mostly from junior faculty ranks, this gap becomes even more evident [3].

With regard to the lower shares of female SA in high-ranked journals compared to medium- or low-ranked journals, a gap in financial means can be discussed as an influencing factor. In most cases, senior authors, as principal investigators, set the course for the success of a study by providing not only their scientific experience but also financial means. Female applicants for third-party funding, however, are on average less likely to receive funding and mostly raise smaller amounts of money than their male counterparts [11]. Concerning governmental grants, the acceptance rates of National Institutes of Health (NIH) grants are comparable between both sexes, but the proportion of female grants awarded still remains noticeably lower [12]**.**

The striking differences in the publication shares of some journals of similar impact factor in this study are comparable to the results of previous studies, e.g., while high rates were described for female FA and SA in *European Radiology*, low rates were found for *Investigative Radiology*, both of them being high-ranked journals [9]. This may in part be explained by different scientific priorities of the journals [9]. For example, the proportion of female authorships is significantly higher in subspecialties for women’s health, such as breast imaging, pediatric radiology, and gynecological imaging, than for interventional subjects [7, 9].

Gender disparity in editorial board composition could be another influencing factor. According to a recently published study on this subject, 81% of large radiological society board members were male, 19% female [13]. While high-ranking (radiological) journals mostly follow a double-blinded review process, 52.1% of the journals in the category “radiology, nuclear medicine, and medical imaging” still used single-blinded review (author identities known by the editor and reviewer) in 2020 [14], which improves acceptance rates for established authors [15]. As those are more likely senior faculty members and thus male, gender disparities in acceptance rates of such journals might exist. Initial submission rates, which we do not know, should also be kept in mind as a probable reason for different gender proportions of female authors between individual journals and different impact areas. It might be that women prefer to submit to medium instead of high impact journals, e.g., due to a lack of confidence, which however would not be logical from an academic career perspective.

Both, the demonstrated general publication gap and the reduced proportions of female senior authors in high-ranked journals are likely to have consequences on female careers. Less authorships in high-ranked journals contribute to lower citation rates of female authors [10] and thus a lower h-index and academic progress. This relation is supported by a study, which showed that after multivariable adjustment of publication counts and NIH funding rates, full professorship among male and female radiologists was not significantly different [11]. As a narrowing of the citation gap for articles of male and female authors in major radiological journals between 1984 and 2014 has been described, further studies should evaluate whether this important development continues [10].

With regards to the higher publication share of male same-sex compared to female same-sex author co-operations and the higher proportions of male SA combinations compared to female SA combinations, one might conclude that junior scientists of both sexes prefer cooperating with a senior male scientist. However, other studies have described a preference of same-sex publishing [9] and collaboration choices might not always be voluntary considering the low proportion of female senior faculty members and the overall low number of female senior authors.

While some journals such as *European Radiology* [16] already strive to evaluate and support female scientists, studies such as this are needed to continuously evaluate the progress of female authors’ representation in Science. Not only is gender parity in Science desirable to support female careers in academic medicine but also because a higher proportion of female authors shifts the focus from the historical orientation of medical research towards the male gender to a better balanced one [7]. Equal representation of women in science on the long-term thus will allow us to also better care for our patients.

This study has some limitations. First, in some cases, it was not possible to assign the author’s gender. Statistics however were exclusively based on the number of total observations, defined as all FA and SA for which gender was successfully assigned. Secondly, the total observations of author names which were successfully assigned to one gender were lower in 2007/2008 than in 2017/2018. This was because in 2007/2008 more first names were only provided as initials and ORCID ID did not exist yet. However, this should have affected male and female authors equally. Also, publication numbers differed between journals (e.g., *n* = 2122 publications in European Radiol in 2017/2018, *n* = 222 in Rofo) which partially resulted in low numbers for the subgroup analysis of individual journals. As each impact area was represented by three journals, low numbers of individual journals however were averaged out in the analysis about the impact areas. Lastly, two of the included journals changed the impact area (MRM between 2007 and 2008 from medium to high; Clin Radiol from low to medium between 2007/2008 and 2017/2018). However, no changes occurred from 2017 to 2018 which would concern the most recent results on author proportions.

In conclusion, the positive trend in the development of female authorships in radiology over the past 10 years is reflected in all impact areas. Female FA and SA increased most in low-ranked journals but are most common in medium-ranked journals. Female SA remain underrepresented in all impact areas, especially in high impact journals. In every impact area, same-sex male authorships remain at least twice as common as all other author collaborations.

## Abbreviations

Acta Radiol: *Acta Radiologica*
AER: Adjusted event rate
AJR: *American Journal of Roentgenology*
AOR: Adjusted odds ratio
Clin Radiol: *Clinical Radiology*
EJR: *European Journal of Radiology*
Eur Radiol: *European Radiology*
FA: First author
Invest Radiol: *Investigative Radiology*
Jpn J Radiol: *Japanese Journal of Radiology*
MRM: *Magnetic Resonance in Medicine*
Rofo: *Fortschritte auf dem Gebiet der Röntgenstrahlen und bildgebenden Verfahren*
SA: Senior author

## Declarations

### Guarantor

The scientific guarantor of this publication is the corresponding author Sarah Keller.

### Statistics and biometry

One of the authors (Ozga A) has significant statistical expertise.

### Methodology

• Retrospective

• Performed at one institution
